# Ethyl 2-amino-4-methyl­thio­phene-3-carboxyl­ate

**DOI:** 10.1107/S2414314621003515

**Published:** 2021-04-09

**Authors:** Ghazala Khanum, Aysha Fatima, Pooja Sharma, S. K. Srivastava, Ray J. Butcher

**Affiliations:** a School of Studies in Chemistry, Jiwaji University, Gwalior 474011, India; b Department of Chemistry, Howard University, 525 College Street NW, Washington DC 20059, USA; Goethe-Universität Frankfurt, Germany

**Keywords:** crystal structure, 2-amino­thio­phene derivative, hydrogen bonding

## Abstract

The title compound, C_8_H_11_NO_2_S, crystallizes with two mol­ecules, *A* and *B*, in the asymmetric unit. Each molecule features an intramolecular N—H⋯O hydrogen bond and the same H atom is also involved in an intermolecular N—H⋯S bond to generate *A* + *B* dimers. Further N—H⋯O hydrogen bonds link the dimers into a [010] chain.

## Abstract 2

The title compound crystallizes with two mol­ecules, *A* and *B*, in the asymmetric unit. Each molecule features an intramolecular N—H⋯O hydrogen bond and the same H atom is also involved in an intermolecular N—H⋯S bond to generate *A* + *B* dimers. Further N—H⋯O hydrogen bonds link the dimers into a [010] chain.

## Graphical abstract

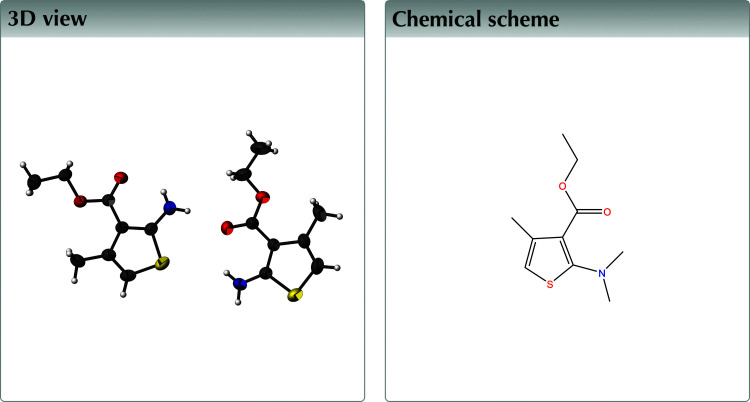

## Structure description

Thio­phene derivatives have been reported to exhibit a broad spectrum of biological properties such as anti-inflammatory, anti­depressant, anti­microbial and anti­convulsant activities (Molvi *et al.*, 2007 ▸; Ashalatha *et al.*, 2007 ▸; Rai *et al.*, 2008 ▸). Thio­phene derivatives are found to be active as allosteric enhancers at the adenosine A1 receptor, which has been linked to anti­arrhythmic and anti­lipolytic activity (Cannito *et al.*,1990 ▸; Lütjens *et al.*, 2003 ▸; Göblyös & Ijzerman, 2009 ▸; Nikolakopoulos *et al.*, 2006 ▸). Thio­phenes also possess properties that are suitable for functional materials, such as field effect transistors (MacDiarmid, 2001 ▸; Kraft, 2001 ▸) and organic light-emitting diodes (Akcelrud, 2003 ▸; Perepichka *et al.*, 2005 ▸) because of their reversible oxidation occurring at low potentials (Nessakh *et al.*, 1995 ▸; van Haare *et al.*, 1995 ▸) and their semiconductor-like behaviour obtained upon *p*-doping (Roncali *et al.*, 2005 ▸).

Many 2-,3-amino­thio­phene derivatives have been prepared so far and the structures of more than 25 of them have been published (see, *e.g.*: Çoruh *et al.*, 2003 ▸; Nirmala *et al.*, 2005 ▸; Bourgeaux & Skene, 2007 ▸; Akkurt *et al.*, 2008 ▸; Zhang & Jiao, 2010 ▸; Ghorab *et al.*, 2012 ▸). Crystal structures of several thio­phenes have been determined in which different functional groups are attached in place of NH_2_ at the 2-position of the ring (Yan & Liu, 2007 ▸; Mukhtar *et al.*, 2012 ▸; de Oliveira *et al.*, 2012 ▸; Mabkhot *et al.*, 2013 ▸; Kaur *et al.*, 2014 ▸). Compounds are known in which the replacement of NH_2_ group by iodine resulted in a cyclo­mer by the association of two monomers through a weak inter­molecular CN⋯I Lewis acid–base inter­action (Moncol *et al.*, 2007 ▸). In the crystal structure of another compound, which is a derivative of piperidine containing amino­thio­phenes, a dimer is formed by the inter­molecular C—H⋯S inter­action between the piperidine and thio­phene rings (Al-Adiwish *et al.*, 2012 ▸).

**Figure 1:**
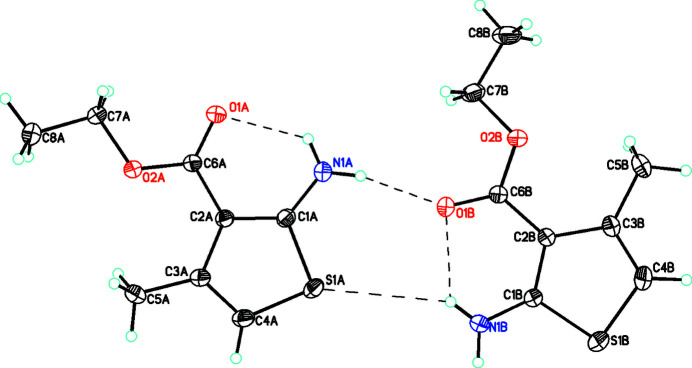
Diagram showing the two mol­ecules *A* and *B* with atom labelling. 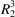 (6) inter­actions involving the NH_2_ and S moieties with a bifurcated hydrogen bond from H1*BA* to S1*A* and O1*B* links the *A* and *B* mol­ecules. Hydrogen bonds are shown with dashed lines. Atomic displacement parameters are at the 30% probability level.

We report herein the synthesis, characterization and crystal structure of the title compound, 2-amino-4-methyl­thio­phene-3-carboxyl­ate (**1**) (Fig. 1 ▸), which crystallizes in the triclinic space group *P* 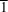 with four mol­ecules in the unit cell (*Z*′ = 2). The two mol­ecules in the asymmetric unit are labelled as *A* and *B*. In both *A* and *B*, the thio­phene ring and the directly attached atoms are all coplanar within experimental error [for *A*: the r.m.s. deviation of the thio­phene moiety is 0.003 (1) Å with N1, C5, and C6 at 0.044 (3), 0.005 (3) and 0.011 (3) Å, respectively; for *B* the r.m.s. deviation is 0.001 (1) Å with N1, C5 and C6 at 0.009 (4), 0.009 (4), and 0.003 (3) Å, respectively]. For *A* the dihedral angle between the thio­phene ring and the NH_2_ substituent is 12.5 (18)° while for the C7, O1 and O2 moiety, this angle is 1.65 (10)°, indicating that this group is almost exactly coplanar with the ring. For *B* the corresponding values are 11 (2) and 2.1 (2)°.

A search for structures containing a 2-amino-thio­phene-3-carboxyl­ate moiety gave 45 hits, two of which are particularly relevant to the current reported structure, *viz*. ethyl 2-amino-4-iso­butyl­thio­phene-3-carboxyl­ate (KIKPIE; Liao *et al.*, 2007 ▸) and ethyl 2-amino-4-phenyl­thio­phen-3-carboxyl­ate (VIWPUM; Dufresne & Skene, 2010 ▸). The only difference between these structures and that of **1** is in the substituent at the 3-position on the ring which are 2-methyl­propyl and phenyl for KIKPIE (Liao *et al.*, 2007 ▸) and VIWPUM (Dufresne & Skene, 2010 ▸). In both cases the metrical parameters are similar as well as the planarity of the substituents.

**Figure 2:**
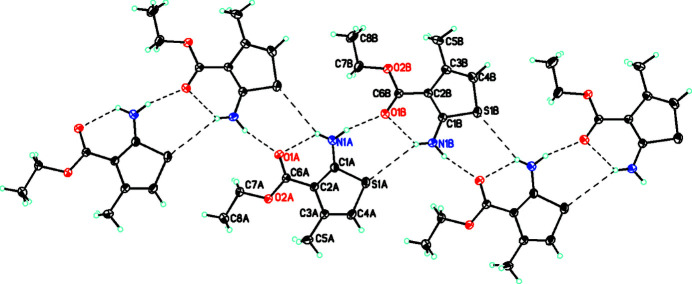
Diagram showing both intra- and inter­molecular hydrogen bonding, which links the mol­ecules into a 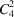 (12) chain in the *b*-axis direction, and 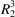 (6) inter­actions involving the NH_2_ and S moieties with a bifurcated hydrogen bond from H1*BA* to S1*A* and O1*B* which links the *A* and *B* mol­ecules. Hydrogen bonds are shown with dashed lines. Atomic displacement parameters are at the 30% probability level.

As far as the packing of the mol­ecules is concerned, there is both intra- and inter­molecular hydrogen bonding. This links the mol­ecules into a 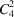 (12) chain in the *b*-axis direction (Etter *et al.*, 1990 ▸). In addition, there are 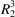 (6) inter­actions involving the NH_2_ and S moieties with a bifurcated hydrogen bond from H1*BA* to S1*A* and O1*B*, which links the *A* and *B* mol­ecules (Table 1 ▸, Figs. 2 ▸ and 3 ▸).

## Synthesis and crystallization

The title compound (ethyl 2-amino-4-methyl­thio­phene-3-carboxyl­ate) (**1**) was prepared by the procedure described in the literature (Zhang *et al.*, 2010 ▸). A mixture of acetone (0.5 mmol) and ethyl­cyano­acetate (0.5 mmol) in absolute ethanol (2 ml) was added to a solution of elemental S (0.5 mmol) and di­ethyl­amine (0.5 mmol) in absolute ethanol (2 ml) and stirred constantly for 3 h at 50°C. The reaction completion was confirmed by using pre-coated silica gel 60 F254 MERCK (20×20 cm). The reaction mixture was quenched with ice-cold water and extracted with ethyl acetate. The organic layer was separated, dried over anhydrous sodium sulfate and concentrated. The crude product was purified using silica gel column chromatography (100–200 mesh) using hexa­ne/ethyl acetate (7:3) mixture solution. Yellow crystals were obtained by slow evaporation of a saturated solution in ethyl acetate and the crystals were used for X-ray diffraction studies. Compound **1**: Yield: (85%). m.p. 76–79°C. **^1^H NMR** (400 MHz, CDCl_3_) δ 6.07 (*s*, 2H), 5.82 (*s*, 1H), 4.29 (*q*, *J* = 7.1 Hz, 2H), 2.28 (*s*, 3H), 1.35 (*t*, *J* = 7.1 Hz, 3H). **^13^C NMR** (400 MHz, CDCl_3_) δ 166.13, 164.17, 136.71, 106.72, 102.85, 59.54, 18.40, 14.40. ESI–MS: *m*/*z* calculated for C_8_H_11_NO_2_S 185.05; found [*M* + H]+ 186.15.

## Refinement

Crystal data, data collection and structure refinement details are summarized in Table 2 ▸.

## Supplementary Material

Crystal structure: contains datablock(s) I. DOI: 10.1107/S2414314621003515/bt4112sup1.cif

Structure factors: contains datablock(s) I. DOI: 10.1107/S2414314621003515/bt4112Isup2.hkl

Supporting information file. DOI: 10.1107/S2414314621003515/bt4112Isup3.cml

CCDC reference: 2074848

Additional supporting information:  crystallographic information; 3D view; checkCIF report

## Figures and Tables

**Figure 3:**
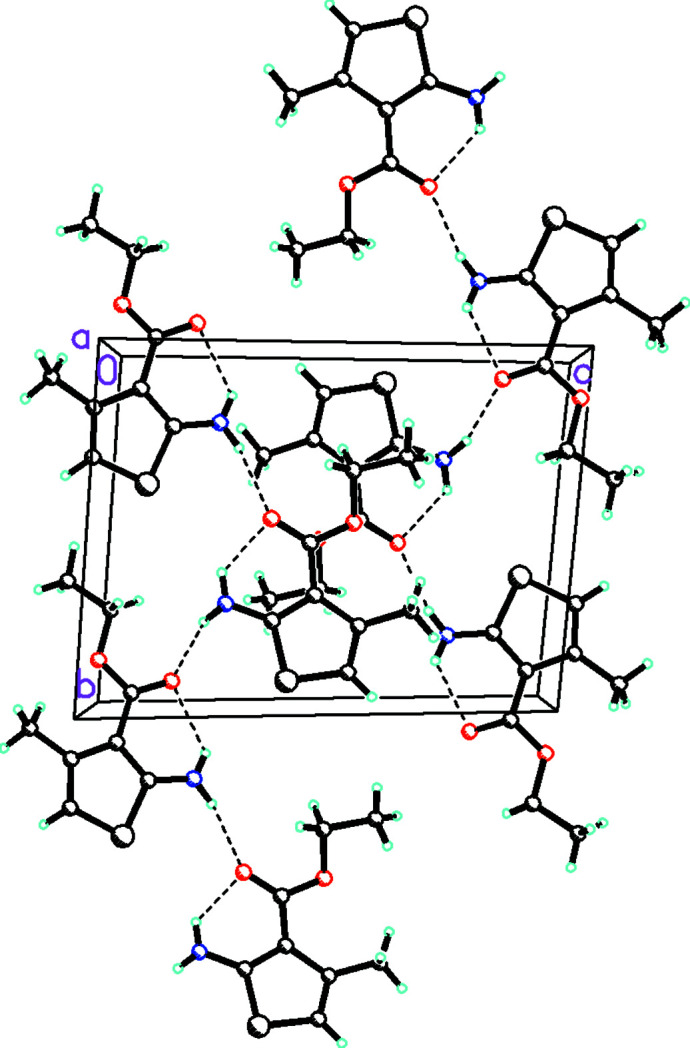
Packing diagram viewed along the *a* axis. Hydrogen bonds are shown with dashed lines.

**Table 1:** Hydrogen-bond geometry (Å, °)

*D*—H⋯*A*	*D*—H	H⋯*A*	*D*⋯*A*	*D*—H⋯*A*
N1*B*—H1*BA*⋯O1*B*	0.82 (3)	2.14 (3)	2.734 (3)	130 (2)
N1*B*—H1*BB*⋯O1*A* ^i^	0.87 (3)	2.10 (3)	2.946 (3)	163 (2)
N1*A*—H1*AA*⋯S1*B* ^ii^	0.83 (3)	3.07 (3)	3.819 (2)	151 (2)
N1*A*—H1*AA*⋯O1*A*	0.83 (3)	2.14 (3)	2.736 (3)	129 (2)
N1*A*—H1*AB*⋯O1*B*	0.86 (3)	2.05 (3)	2.897 (3)	165 (2)
C7*A*—H7*AA*⋯S1*A* ^iii^	0.97	3.02	3.736 (3)	131

Symmetry codes: (i) 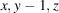 ; (ii) 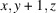 ; (iii) 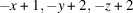 .

**Table 2:** Experimental details

Crystal data	
Chemical formula	C_8_H_11_NO_2_S
*M*_r_	185.24
Crystal system, space group	Triclinic, *P* 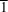
Temperature (K)	293
*a*, *b*, *c* (Å)	7.664 (3), 9.876 (3), 13.018 (5)
α, β, γ (°)	91.602 (12), 104.301 (13), 101.729 (13)
*V* (Å^3^)	931.7 (6)
*Z*	4
Radiation type	Mo *K*α
μ (mm^−1^)	0.31
Crystal size (mm)	0.48 × 0.35 × 0.12

Data collection	
Diffractometer	Bruker APEXII CCD
Absorption correction	Multi-scan (*SADABS*; Sheldrick, 1996 ▸)
*T*_min_, *T*_max_	0.565, 0.747
No. of measured, independent and observed [*I* > 2σ(*I*)] reflections	27589, 5636, 3845
*R*_int_	0.062
(sin θ/λ)_max_ (Å^−1^)	0.714

Refinement	
*R*[*F* ^2^ > 2σ(*F* ^2^)], *wR*(*F* ^2^), *S*	0.057, 0.168, 1.03
No. of reflections	5636
No. of parameters	237
H-atom treatment	H atoms treated by a mixture of independent and constrained refinement
Δρ_max_, Δρ_min_ (e Å^−3^)	0.62, −0.39

Computer programs: *APEX2* (Bruker, 2005 ▸), *SAINT* (Bruker, 2002 ▸), *SHELXT* (Sheldrick 2015 ▸a), *SHELXL2018/3* (Sheldrick, 2015 ▸b) and *SHELXTL* (Sheldrick 2008 ▸).

## full crystallographic data

### Crystal data

**Table:**

C_8_H_11_NO_2_S	*Z* = 4
*M**_r_* = 185.24	*F*(000) = 392
Triclinic, *P*1	*D*_x_ = 1.321 Mg m^−^^3^
*a* = 7.664 (3) Å	Mo *K*α radiation, λ = 0.71073 Å
*b* = 9.876 (3) Å	Cell parameters from 9004 reflections
*c* = 13.018 (5) Å	θ = 2.6–30.7°
α = 91.602 (12)°	µ = 0.31 mm^−^^1^
β = 104.301 (13)°	*T* = 293 K
γ = 101.729 (13)°	Plate, colourless
*V* = 931.7 (6) Å^3^	0.48 × 0.35 × 0.12 mm

### Data collection

**Table:**

Bruker APEXII CCD diffractometer	3845 reflections with *I* > 2σ(*I*)
φ and ω scans	*R*_int_ = 0.062
Absorption correction: multi-scan (SADABS; Sheldrick, 1996)	θ_max_ = 30.5°, θ_min_ = 2.1°
*T*_min_ = 0.565, *T*_max_ = 0.747	*h* = −10→10
27589 measured reflections	*k* = −14→14
5636 independent reflections	*l* = −18→18

### Refinement

**Table:**

Refinement on *F*^2^	Primary atom site location: structure-invariant direct methods
Least-squares matrix: full	Secondary atom site location: difference Fourier map
*R*[*F*^2^ > 2σ(*F*^2^)] = 0.057	Hydrogen site location: mixed
*wR*(*F*^2^) = 0.168	H atoms treated by a mixture of independent and constrained refinement
*S* = 1.03	*w* = 1/[σ^2^(*F*_o_^2^) + (0.0812*P*)^2^ + 0.2238*P*] where *P* = (*F*_o_^2^ + 2*F*_c_^2^)/3
5636 reflections	(Δ/σ)_max_ < 0.001
237 parameters	Δρ_max_ = 0.62 e Å^−^^3^
0 restraints	Δρ_min_ = −0.39 e Å^−^^3^

### Special details

**Table:**

Geometry. All esds (except the esd in the dihedral angle between two l.s. planes) are estimated using the full covariance matrix. The cell esds are taken into account individually in the estimation of esds in distances, angles and torsion angles; correlations between esds in cell parameters are only used when they are defined by crystal symmetry. An approximate (isotropic) treatment of cell esds is used for estimating esds involving l.s. planes.
Refinement. The structure was solved with *SHELXT* (Sheldrick, 2015*a*) and refined with *SHELXL2018*/3 (Sheldrick 2015*b*). The amine hydrogen atoms were refined isotropically while the C-bound H atoms were included in calculated positions and treated as riding, with C—H = 0.95–0.98 Å, and with 1.2*U*_eq_(C) for H atoms.

### Fractional atomic coordinates and isotropic or equivalent isotropic displacement parameters (Å^2^)

**Table:**

	*x*	*y*	*z*	*U*_iso_*/*U*_eq_	
S1A	0.57912 (8)	0.63040 (5)	0.90873 (4)	0.05608 (17)	
O1A	0.5956 (2)	1.07513 (14)	0.82938 (11)	0.0598 (4)	
O2A	0.77241 (18)	1.13164 (13)	0.99478 (10)	0.0469 (3)	
N1A	0.4678 (3)	0.7995 (2)	0.76263 (15)	0.0611 (5)	
H1AA	0.478 (4)	0.878 (3)	0.741 (2)	0.074 (8)*	
H1AB	0.420 (4)	0.728 (3)	0.718 (2)	0.067 (7)*	
C1A	0.5667 (3)	0.79097 (18)	0.86174 (15)	0.0434 (4)	
C2A	0.6638 (2)	0.89702 (17)	0.93919 (13)	0.0394 (4)	
C3A	0.7481 (2)	0.84624 (19)	1.03824 (14)	0.0429 (4)	
C4A	0.7121 (3)	0.7062 (2)	1.03180 (17)	0.0535 (5)	
H4AA	0.754788	0.655468	1.088165	0.064*	
C5A	0.8619 (3)	0.9325 (2)	1.13746 (16)	0.0580 (5)	
H5AA	0.899385	0.872987	1.191859	0.087*	
H5AB	0.790195	0.990278	1.160998	0.087*	
H5AC	0.969117	0.989811	1.123237	0.087*	
C6A	0.6715 (2)	1.03954 (17)	0.91494 (14)	0.0400 (4)	
C7A	0.7865 (3)	1.27623 (18)	0.97679 (16)	0.0501 (4)	
H7AA	0.664931	1.297311	0.957187	0.060*	
H7AB	0.845948	1.299239	0.919940	0.060*	
C8A	0.8989 (3)	1.3566 (2)	1.07851 (18)	0.0607 (5)	
H8AA	0.910249	1.454059	1.070404	0.091*	
H8AB	1.019220	1.335704	1.096380	0.091*	
H8AC	0.839418	1.331695	1.134225	0.091*	
S1B	0.35148 (12)	0.08122 (6)	0.57884 (5)	0.0809 (3)	
O1B	0.3541 (2)	0.53445 (14)	0.63773 (11)	0.0620 (4)	
O2B	0.2205 (2)	0.52307 (14)	0.46392 (11)	0.0538 (3)	
N1B	0.4376 (3)	0.2976 (2)	0.72151 (14)	0.0639 (5)	
H1BA	0.451 (3)	0.381 (3)	0.7357 (18)	0.057 (7)*	
H1BB	0.484 (4)	0.242 (3)	0.765 (2)	0.076 (8)*	
C1B	0.3661 (3)	0.25098 (19)	0.61927 (15)	0.0474 (4)	
C2B	0.2967 (3)	0.32322 (18)	0.53404 (13)	0.0425 (4)	
C3B	0.2320 (3)	0.2374 (2)	0.43493 (16)	0.0564 (5)	
C4B	0.2549 (5)	0.1078 (3)	0.4495 (2)	0.0824 (8)	
H4BA	0.221258	0.038177	0.394211	0.099*	
C5B	0.1488 (4)	0.2818 (3)	0.32835 (17)	0.0748 (7)	
H5BA	0.121058	0.205434	0.275478	0.112*	
H5BB	0.234517	0.357479	0.311217	0.112*	
H5BC	0.037419	0.311113	0.329922	0.112*	
C6B	0.2961 (3)	0.46767 (18)	0.55163 (14)	0.0424 (4)	
C7B	0.2082 (3)	0.6659 (2)	0.47361 (19)	0.0609 (5)	
H7BA	0.330234	0.725322	0.498848	0.073*	
H7BB	0.136218	0.679395	0.523229	0.073*	
C8B	0.1157 (4)	0.6988 (3)	0.3643 (2)	0.0809 (8)	
H8BA	0.089268	0.789501	0.367982	0.121*	
H8BB	0.002774	0.631283	0.336545	0.121*	
H8BC	0.195895	0.696714	0.318332	0.121*	

### Atomic displacement parameters (Å^2^)

**Table:**

	*U*^11^	*U*^22^	*U*^33^	*U*^12^	*U*^13^	*U*^23^
S1A	0.0707 (4)	0.0344 (2)	0.0629 (3)	0.0106 (2)	0.0173 (3)	0.0055 (2)
O1A	0.0810 (10)	0.0432 (7)	0.0456 (7)	0.0181 (7)	−0.0054 (7)	0.0073 (6)
O2A	0.0547 (8)	0.0354 (6)	0.0441 (7)	0.0084 (5)	0.0020 (6)	0.0059 (5)
N1A	0.0846 (14)	0.0431 (9)	0.0456 (9)	0.0128 (9)	0.0003 (9)	−0.0027 (8)
C1A	0.0497 (10)	0.0369 (8)	0.0453 (9)	0.0108 (7)	0.0141 (8)	0.0038 (7)
C2A	0.0404 (9)	0.0366 (8)	0.0414 (8)	0.0088 (7)	0.0103 (7)	0.0065 (6)
C3A	0.0425 (9)	0.0434 (9)	0.0450 (9)	0.0116 (7)	0.0128 (7)	0.0109 (7)
C4A	0.0631 (12)	0.0458 (10)	0.0543 (11)	0.0166 (9)	0.0144 (9)	0.0176 (8)
C5A	0.0635 (13)	0.0579 (12)	0.0444 (10)	0.0124 (10)	−0.0012 (9)	0.0109 (9)
C6A	0.0428 (9)	0.0378 (8)	0.0400 (8)	0.0115 (7)	0.0091 (7)	0.0055 (6)
C7A	0.0621 (12)	0.0345 (8)	0.0500 (10)	0.0116 (8)	0.0062 (9)	0.0055 (7)
C8A	0.0653 (13)	0.0454 (10)	0.0605 (13)	0.0052 (9)	0.0021 (10)	−0.0021 (9)
S1B	0.1329 (7)	0.0398 (3)	0.0631 (4)	0.0278 (3)	0.0053 (4)	0.0040 (2)
O1B	0.0879 (11)	0.0431 (7)	0.0452 (7)	0.0163 (7)	−0.0015 (7)	−0.0043 (6)
O2B	0.0696 (9)	0.0449 (7)	0.0446 (7)	0.0190 (6)	0.0046 (6)	0.0083 (6)
N1B	0.0985 (16)	0.0499 (10)	0.0380 (8)	0.0265 (10)	−0.0011 (9)	0.0056 (8)
C1B	0.0601 (11)	0.0383 (8)	0.0419 (9)	0.0129 (8)	0.0077 (8)	0.0040 (7)
C2B	0.0492 (10)	0.0395 (8)	0.0360 (8)	0.0107 (7)	0.0052 (7)	0.0015 (6)
C3B	0.0707 (13)	0.0495 (10)	0.0404 (9)	0.0104 (9)	0.0015 (9)	−0.0040 (8)
C4B	0.128 (2)	0.0480 (12)	0.0570 (13)	0.0180 (14)	0.0015 (14)	−0.0132 (10)
C5B	0.1004 (19)	0.0743 (15)	0.0386 (10)	0.0216 (14)	−0.0035 (11)	−0.0068 (10)
C6B	0.0463 (9)	0.0398 (8)	0.0399 (8)	0.0107 (7)	0.0074 (7)	0.0050 (7)
C7B	0.0672 (13)	0.0447 (10)	0.0700 (14)	0.0180 (9)	0.0105 (11)	0.0160 (9)
C8B	0.0828 (18)	0.0716 (16)	0.0840 (18)	0.0226 (14)	0.0055 (14)	0.0366 (14)

### Geometric parameters (Å, º)

**Table:**

S1A—C4A	1.727 (2)	S1B—C4B	1.715 (3)
S1A—C1A	1.7277 (19)	S1B—C1B	1.716 (2)
O1A—C6A	1.220 (2)	O1B—C6B	1.217 (2)
O2A—C6A	1.330 (2)	O2B—C6B	1.333 (2)
O2A—C7A	1.440 (2)	O2B—C7B	1.437 (2)
N1A—C1A	1.340 (3)	N1B—C1B	1.337 (3)
N1A—H1AA	0.83 (3)	N1B—H1BA	0.82 (3)
N1A—H1AB	0.86 (3)	N1B—H1BB	0.87 (3)
C1A—C2A	1.386 (3)	C1B—C2B	1.389 (2)
C2A—C6A	1.444 (2)	C2B—C6B	1.440 (2)
C2A—C3A	1.445 (2)	C2B—C3B	1.443 (3)
C3A—C4A	1.350 (3)	C3B—C4B	1.339 (3)
C3A—C5A	1.495 (3)	C3B—C5B	1.495 (3)
C4A—H4AA	0.9300	C4B—H4BA	0.9300
C5A—H5AA	0.9600	C5B—H5BA	0.9600
C5A—H5AB	0.9600	C5B—H5BB	0.9600
C5A—H5AC	0.9600	C5B—H5BC	0.9600
C7A—C8A	1.492 (3)	C7B—C8B	1.502 (3)
C7A—H7AA	0.9700	C7B—H7BA	0.9700
C7A—H7AB	0.9700	C7B—H7BB	0.9700
C8A—H8AA	0.9600	C8B—H8BA	0.9600
C8A—H8AB	0.9600	C8B—H8BB	0.9600
C8A—H8AC	0.9600	C8B—H8BC	0.9600
			
C4A—S1A—C1A	91.37 (9)	C4B—S1B—C1B	91.28 (11)
C6A—O2A—C7A	117.21 (14)	C6B—O2B—C7B	117.81 (16)
C1A—N1A—H1AA	116.2 (19)	C1B—N1B—H1BA	116.5 (17)
C1A—N1A—H1AB	122.2 (17)	C1B—N1B—H1BB	118.1 (18)
H1AA—N1A—H1AB	120 (2)	H1BA—N1B—H1BB	124 (2)
N1A—C1A—C2A	128.91 (17)	N1B—C1B—C2B	128.53 (18)
N1A—C1A—S1A	119.95 (15)	N1B—C1B—S1B	120.36 (15)
C2A—C1A—S1A	111.12 (14)	C2B—C1B—S1B	111.12 (14)
C1A—C2A—C6A	119.56 (16)	C1B—C2B—C6B	119.66 (16)
C1A—C2A—C3A	112.69 (15)	C1B—C2B—C3B	112.43 (17)
C6A—C2A—C3A	127.75 (16)	C6B—C2B—C3B	127.92 (17)
C4A—C3A—C2A	111.36 (17)	C4B—C3B—C2B	111.01 (19)
C4A—C3A—C5A	122.25 (18)	C4B—C3B—C5B	122.6 (2)
C2A—C3A—C5A	126.40 (16)	C2B—C3B—C5B	126.34 (19)
C3A—C4A—S1A	113.46 (15)	C3B—C4B—S1B	114.16 (17)
C3A—C4A—H4AA	123.3	C3B—C4B—H4BA	122.9
S1A—C4A—H4AA	123.3	S1B—C4B—H4BA	122.9
C3A—C5A—H5AA	109.5	C3B—C5B—H5BA	109.5
C3A—C5A—H5AB	109.5	C3B—C5B—H5BB	109.5
H5AA—C5A—H5AB	109.5	H5BA—C5B—H5BB	109.5
C3A—C5A—H5AC	109.5	C3B—C5B—H5BC	109.5
H5AA—C5A—H5AC	109.5	H5BA—C5B—H5BC	109.5
H5AB—C5A—H5AC	109.5	H5BB—C5B—H5BC	109.5
O1A—C6A—O2A	121.81 (16)	O1B—C6B—O2B	121.96 (17)
O1A—C6A—C2A	124.29 (16)	O1B—C6B—C2B	124.56 (17)
O2A—C6A—C2A	113.89 (15)	O2B—C6B—C2B	113.46 (16)
O2A—C7A—C8A	106.65 (16)	O2B—C7B—C8B	106.1 (2)
O2A—C7A—H7AA	110.4	O2B—C7B—H7BA	110.5
C8A—C7A—H7AA	110.4	C8B—C7B—H7BA	110.5
O2A—C7A—H7AB	110.4	O2B—C7B—H7BB	110.5
C8A—C7A—H7AB	110.4	C8B—C7B—H7BB	110.5
H7AA—C7A—H7AB	108.6	H7BA—C7B—H7BB	108.7
C7A—C8A—H8AA	109.5	C7B—C8B—H8BA	109.5
C7A—C8A—H8AB	109.5	C7B—C8B—H8BB	109.5
H8AA—C8A—H8AB	109.5	H8BA—C8B—H8BB	109.5
C7A—C8A—H8AC	109.5	C7B—C8B—H8BC	109.5
H8AA—C8A—H8AC	109.5	H8BA—C8B—H8BC	109.5
H8AB—C8A—H8AC	109.5	H8BB—C8B—H8BC	109.5
			
C4A—S1A—C1A—N1A	−177.88 (18)	C4B—S1B—C1B—N1B	−179.6 (2)
C4A—S1A—C1A—C2A	0.54 (15)	C4B—S1B—C1B—C2B	0.33 (19)
N1A—C1A—C2A—C6A	−2.3 (3)	N1B—C1B—C2B—C6B	−0.3 (3)
S1A—C1A—C2A—C6A	179.41 (13)	S1B—C1B—C2B—C6B	179.78 (15)
N1A—C1A—C2A—C3A	177.8 (2)	N1B—C1B—C2B—C3B	179.7 (2)
S1A—C1A—C2A—C3A	−0.5 (2)	S1B—C1B—C2B—C3B	−0.3 (2)
C1A—C2A—C3A—C4A	0.1 (2)	C1B—C2B—C3B—C4B	0.0 (3)
C6A—C2A—C3A—C4A	−179.77 (18)	C6B—C2B—C3B—C4B	180.0 (2)
C1A—C2A—C3A—C5A	−179.96 (18)	C1B—C2B—C3B—C5B	179.7 (2)
C6A—C2A—C3A—C5A	0.2 (3)	C6B—C2B—C3B—C5B	−0.3 (4)
C2A—C3A—C4A—S1A	0.3 (2)	C2B—C3B—C4B—S1B	0.2 (3)
C5A—C3A—C4A—S1A	−179.62 (16)	C5B—C3B—C4B—S1B	−179.5 (2)
C1A—S1A—C4A—C3A	−0.52 (17)	C1B—S1B—C4B—C3B	−0.3 (3)
C7A—O2A—C6A—O1A	0.9 (3)	C7B—O2B—C6B—O1B	−0.2 (3)
C7A—O2A—C6A—C2A	−179.88 (16)	C7B—O2B—C6B—C2B	178.22 (17)
C1A—C2A—C6A—O1A	0.7 (3)	C1B—C2B—C6B—O1B	0.4 (3)
C3A—C2A—C6A—O1A	−179.46 (18)	C3B—C2B—C6B—O1B	−179.5 (2)
C1A—C2A—C6A—O2A	−178.48 (15)	C1B—C2B—C6B—O2B	−177.98 (17)
C3A—C2A—C6A—O2A	1.4 (3)	C3B—C2B—C6B—O2B	2.1 (3)
C6A—O2A—C7A—C8A	177.71 (17)	C6B—O2B—C7B—C8B	−179.07 (18)

### Hydrogen-bond geometry (Å, º)

**Table:**

*D*—H···*A*	*D*—H	H···*A*	*D*···*A*	*D*—H···*A*
N1*B*—H1*BA*···O1*B*	0.82 (3)	2.14 (3)	2.734 (3)	130 (2)
N1*B*—H1*BB*···O1*A*^i^	0.87 (3)	2.10 (3)	2.946 (3)	163 (2)
N1*A*—H1*AA*···S1*B*^ii^	0.83 (3)	3.07 (3)	3.819 (2)	151 (2)
N1*A*—H1*AA*···O1*A*	0.83 (3)	2.14 (3)	2.736 (3)	129 (2)
N1*A*—H1*AB*···O1*B*	0.86 (3)	2.05 (3)	2.897 (3)	165 (2)
C7*A*—H7*AA*···S1*A*^iii^	0.97	3.02	3.736 (3)	131

Symmetry codes: (i) *x*, *y*−1, *z*; (ii) *x*, *y*+1, *z*; (iii) −*x*+1, −*y*+2, −*z*+2.
